# Comprehensive quantitative analysis of muscle cross-sectional area and intramuscular fat infiltration of hip using modified DIXON MRI based on chemical shift encoding technique

**DOI:** 10.3389/fragi.2026.1726714

**Published:** 2026-03-26

**Authors:** Dezhao Jia, Ming Wang, Hong Yu, Yongzhi Zhang, Yi Wang, Yiming Sun, Mengfei Wu

**Affiliations:** 1 Department of Radiology, Hebei General Hospital, Shijiazhuang, Hebei, China; 2 Department of Radiology, Hebei Medical University Third Hospital, Shijiazhuang, Hebei, China

**Keywords:** chemical shift encoding, cross sectional area, hip muscles, intramuscular fat infiltration, modified DIXON sequence

## Abstract

**Purpose:**

Noninvasive quantitative assessment of hip musculature is critically needed, as the structural integrity of hip muscles and the degree of intramuscular fat infiltration are essential determinants of hip stability and function. This study employed a modified DIXON of magnetic resonance imaging (MRI) technique to evaluate the cross sectional area (CSA) and intramuscular fat fraction (IMFF) of hip muscles. We analyzed factors influencing changes in hip muscle structure and intramuscular fat infiltration with the aim of establishing a comprehensive map of hip muscle characteristics.

**Methods:**

This cross-sectional study enrolled participants from the health examination center of our research institution. All participants underwent 3.0T MRI with mDIXON Quant sequence. The CSA and IMFF of 10 muscles of hip were semi-automatically delineated and measured at four predefined anatomical levels. the effects of sex, age, and laterality on hip muscle CSA and IMFF were investigated. Multivariate analysis was also performed to identify factors influencing IMFF in the hip.

**Results:**

A total of 177 participants (100 males, 77 females; age range: 18–80 years) were included. The mean age was 52.70 ± 14.36 years for males and 51.58 ± 14.85 years for females. Compared with females, males exhibited significantly smaller subcutaneous fat area (SFA)/subcutaneous fat thickness (SFT) (p < 0.001) and a larger proximal femoral area (PFA) in the hip (p < 0.001). Additionally, males demonstrated larger CSA in all muscles (p < 0.001) and lower IMFF in most muscles (p ≤ 0.001) of hip. With advancing age, the CSA of all 10 hip muscles progressively decreased, while IMFF increased. Laterality had minimal influence on hip muscle CSA and IMFF. Multivariate analyses showed that age as the predominant factor influencing IMFF.

**Conclusion:**

The mDIXON QUANT sequence enables noninvasive quantification of CSA and IMFF of hip muscles, with age identified as a significant influencing factor. This technique holds promise for establishing normative reference data on hip muscle CSA and IMFF in healthy populations, thereby enhancing the understanding of degenerative changes and aiding in clinical decision-making for surgical planning and postoperative rehabilitation guidance.

## 1 Introduction

The hip muscle group constitutes a critical functional unit for maintaining upright posture, hip joint stability, and energy metabolism in humans. Dynamic changes in its morphology and composition are closely associated with osteoarthritis, sarcopenia, and sports injuries (Lang et al., 2010; Zhang et al., 2023). Muscle cross sectional area (CSA) and intramuscular fat fraction (IMFF) serve as critical metrics for evaluating muscle mass and function. Quantitative assessment of CSA and IMFF not only untangles the dynamic evolution of muscle morphology-function relationships but also provides a pivotal entry point for investigating the pathological mechanisms underlying degenerative diseases.

Traditionally, muscle biopsy remains the histological “gold standard”; however, its invasive nature and sampling limitations impede its utility for dynamic clinical monitoring. Consequently, the development of precise, noninvasive imaging-based quantitative techniques has emerged as a key focus in musculoskeletal research. Magnetic resonance imaging (MRI), owing to its superior soft tissue delineation, has become the modality of choice for assessing muscle morphology and composition. Chemical shift encoding-based techniques, such as the DIXON sequence and its derivatives (e.g., mDIXON Quant, IDEAL-IQ), leverage water-fat separation principles to generate quantitative fat fraction maps, thereby enabling accurate analysis of intramuscular fat content (Belzunce et al., 2022).

Current MRI-based studies on hip musculature have provided valuable foundational insights. Notably, research has predominantly focused on limb muscles (e.g., quadriceps) or specific hip muscle groups, particularly the abductors and gluteal muscles, exploring differences related to age and sex (Marcon et al., 2016; Belzunce et al., 2022). However, a comprehensive and simultaneous quantification of a broad range of hip muscles—including flexors, adductors, and stabilizers—in healthy populations remains limited. This gap has resulted in a lack of systematic, age- and sex-stratified normative reference data for muscle CSA and IMFF across the hip region, thereby hindering cross-study comparability and the clinical translation of standardized muscle degeneration assessment frameworks.

Establishing normative, age- and sex-stratified reference data for hip muscle morphology and composition is of paramount clinical importance. Age-related sarcopenia and fat infiltration are key determinants of musculoskeletal decline, affecting joint stability, mobility, and metabolic health. However, distinguishing physiological aging from pathological degeneration requires robust baseline data across the adult lifespan. Furthermore, significant sex differences in muscle mass, fat distribution, and hormonal regulation necessitate separate reference standards. Such detailed stratification is crucial for accurate clinical assessment, preoperative risk evaluation, and the development of personalized rehabilitation protocols in conditions ranging from osteoarthritis to hip fractures.

This study systematically investigated age- and sex-related variations in hip muscle CSA and IMFF in a healthy Asian population, while also examining bilateral differences. Our work advances the field in several key aspects: by systematically assessing ten key hip muscles to provide a more holistic anatomical map; by analyzing a larger cohort to enhance the statistical power and generalizability of normative data; and by implementing a multi-level anatomical averaging protocol for CSA and IMFF to reduce slice selection bias and obtain more representative whole muscle estimates.

The primary objectives of this study were to establish a robust chemical shift-encoded quantitative MRI protocol for the hip using the mDIXON Quant sequence, and to generate stratified normative reference data for muscle CSA and IMFF across adult age decades and between sexes. Secondary objectives included that analyzing the effects of age, sex laterality, and exploring anatomical correlations between muscle parameters and local body composition metrics. By establishing this detailed cross-sectional baseline in healthy individuals, we aim to infer physiological progression patterns of hip muscle degeneration, thereby providing a critical data foundation for defining pathological thresholds, supporting preoperative assessment, and guiding personalized rehabilitation strategies.

## 2 Materials and methods

### 2.1 Ethical approval and study population

This study was conducted as a cross-sectional investigation and received approval from the Ethics Committee of the Third Hospital of Hebei Medical University (IRB approval number: W2023-007-1). A total of 177 healthy participants (100 males and 77 females) were enrolled between 2023 and 2024. All study participants were recruited from the health examination center of our hospital, with Asian ethnicity and right-handedness confirmed. The use of self-reported handedness served as a pragmatic surrogate for general limb dominance in this population-based study. Inclusion criteria comprised: age between 18 and 80 years; no participation in organized physical exercise (defined as <2 h of weekly exercise), body mass index (BMI) between the 10th and 90th percentiles, absence of metabolic disorders, and no history of hip surgery, developmental deformities, trauma, neoplasms, or systemic inflammation.

### 2.2 MRI acquisition

All MRI examinations were performed on a 3.0T Philips Ingenia CX (Phillips, Amsterdam, Netherlands) scanner using a 32-channel torso coil. The mDIXON Quant sequence was employed for chemical shift encoding-based water-fat separation and quantification. The acquisition parameters were as follows: axial = fast field echo; repetition time = 9.2 ms; echo time (TE1) = 1.2 ms; ΔTE = 0.7 ms, with subsequent echoes at TE2 = 1.9, TE3 = 2.6, TE4 = 3.3, TE5 = 4.0, and TE6 = 4.7 ms; slice thickness = 3mm; field of view = 320 × 419 × 135mm; voxel = 1.8 × 1.2 × 3mm; Acquisition matrix size = 288 × 194 mm; flip angle = 3°; NSA = 2. Imaging coverage extended axially from the anterior superior iliac spine to the lower edge of the lesser trochanter, comprising 60 slices to ensure complete visualization of all target hip muscles. The sequence simultaneously reconstructs water-only, fat-only, in-phase, out-of-phase, T2*, and fat fraction maps.

### 2.3 Image analysis and measurements

The CSA and IMFF of 10 muscles were semi-automatically quantified using the traditional manual region-of-interest (ROI) delineation method on the IntelliSpace Portal Version seven workstation. The analyzed muscles included: tensor fasciae latae (TFL), gluteus minimus (GMin), gluteus medius (GMed), gluteus maximus (GMax), sartorius, iliopsoas (ILPS), rectus femoris (RF), pectineus (PEC), internal obturator (OR), and vastus lateralis (VL).

**FIGURE 1:**
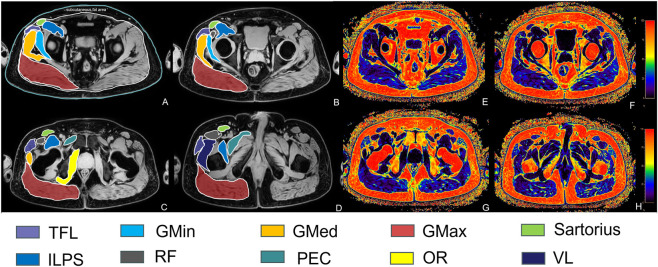
Axial images at four anatomical levels showing the segmentation strategy for ROI analysis. **(A–D)** displays water-only images, and **(E–H)** displays the corresponding fat fraction color maps. **(A,E)** Acetabular level; **(B,F)** Femoral head level; **(C,G)** Femoral neck level; **(D,H)** Lesser trochanter level. Regions of interest (ROIs) were manually delineated for the following muscles and areas: TFL; GMin; GMed; GMax; sartorius; ILPS; RF; PEC; OR and VL and SFA. TFL, tensor fasciae latae; GMin, gluteus minimus; GMed, gluteus medius; GMax, gluteus maximus; ILPS, iliopsoas; RF, rectus femoris; PEC, pectineus; OR, internal obturator; VL, vastus lateralis; SFA, subcutaneous fat area.

The semi-automated process involved two steps: First, an experienced musculoskeletal radiologist (Observer A, with 4 years of experience) manually delineated the ROI for each muscle on the axial water-only images at four predefined anatomical levels: 1) acetabular level, 2) femoral head level, 3) femoral neck level, and 4) lesser trochanter level. This step ensured precise anatomical definition of muscle boundaries. Second, the manually defined ROI was automatically copied onto the coregistered fat fraction map. The workstation then calculated the CSA (mm^2^) and the mean IMFF (%) within each ROI. The final CSA and IMFF value for each muscle was computed as the average of measurements obtained across all anatomical levels where the muscle was distinctly visible (Figure 1).

Additional measurements included:

1. Subcutaneous fat area (SFA) was measured at the acetabular level. Subcutaneous fat thickness (SFT) was measured on the same slice by drawing a line perpendicular to the skin surface from the outer skin border to the superficial fascia of the GMax at its posterior margin, where the subcutaneous layer was thickest.
2. Proximal femoral area (PFA), defined as the total CSA of the femur, was measured at the level of the lower edge of the lesser trochanter, serving as a local anatomical reference for bone size.

All initial measurements were performed by Observer A. For quality control, 10% of cases were randomly selected for re-measurement of parameters of GMax at the acetabular level by a senior musculoskeletal radiologist (Observer B) with over 10 years of experience. Interobserver agreement was assessed using intraclass correlation coefficient (ICC).

### 2.4 Data analysis

All statistical analyses were performed using IBM SPSS Statistics for Windows, version 29 (IBM Corp., Armonk, N.Y., USA). Normality of data was assessed using the Shapiro-Wilk test (α = 0.05). ICC was employed for consistency analysis, with ICC >0.75 considered good agreement. Intergroup comparisons were conducted using the Mann-Whitney U test (for sex-), Kruskal–Wallis test with Bonferroni correction (for age-), and Wilcoxon signed-rank test (for bilateral asymmetry). The correlation analysis was conducted using the Spearman correlation method. Multivariate analysis was performed using generalized estimating equations (GEE). Data are presented as mean ± standard deviation (SD), with a significance threshold of p < 0.05.

## 3 Results

The clinical characteristics of the participants in this study are presented in Table 1. The ICC between Observer A and Observer B was 0.978, indicating excellent agreement.

**TABLE 1:** Comparison of baseline characteristics and measurement Indices of hip muscle between male and female.

Factors	Male (n = 100)		Female (n = 77)		*P*
	Mean	SD	Mean	SD	
Age (years)	52.70	14.36	51.58	14.85	0.612
SFA (mm^2^)	13716.54	4159.94	19568.80	5805.51	**0.000**
SFT (mm)	14.88	3.90	22.73	7.15	**0.000**
PFA (mm^2^)	674.28	129.78	552.60	101.26	**0.000**

SFA, subcutaneous fat area; SFT, subcutaneous fat thickness; PFA, proximal femoral area; CSA, cross sectional area. Bold font indicates P < 0.05.

### 3.1 Sex differences in CSA and fat infiltration of hip muscles

**FIGURE 2:**
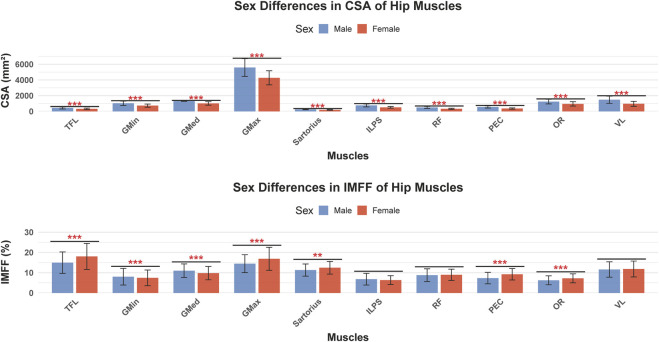
Comparison of hip muscle CSA and IMFF between males and females (*p < 0.05, **p < 0.01, ***p < 0.001). CSA,cross sectional area; IMFF, intramuscular fat fraction; TFL,tensor fasciae latae; GMin, gluteus minimus; GMed, gluteus medius; GMax, gluteus maximus; ILPS, iliopsoas; RF, rectus femoris; PEC, pectineus; OR, internal obturator; VL, vastus lateralis.

Significantly smaller SFA and SFT was observed in males compared to females (p < 0.001), and the proximal area of the femur was larger in males than in females (p < 0.001). Males exhibited larger CSA of muscle across all 10 measured muscle groups (p < 0.001), with notable differences in the CSA of GMax (5,588.14 ± 1,139.46 mm^2^ in males vs. 4,272.48 ± 898.51 mm^2^ in females, p < 0.001). Furthermore, males demonstrated lower IMFF in most muscles compared to females, with significant differences observed in TFL, GMin, sartorius, PEC, and OR (p < 0.05). However, IMFF in the GMax and GMed was more severe in males than in females (Figure 2; Table 2).

**TABLE 2:** Analysis of sex differences in CSA and IMFF of hip muscle.

Muscle	Gender	CSA (mm^2^)		*P* (Male vs. Female)	IMFF(%)		*P* (Male vs. Female)
		Mean	SD		Mean	SD	
TFL	Male	436.08	128.06	**0.000**	14.96	5.29	**0.000**
	Female	322.39	91.36	​	18.05	6.43	​
GMin	Male	1023.34	279.59	**0.000**	8.02	4.12	**0.000**
	Female	726.44	193.39	​	7.44	3.88	​
GMed	Male	1311.16	31.57	**0.000**	10.99	3.35	**0.000**
	Female	1010.43	225.08	​	9.78	3.31	​
GMax	Male	5588.14	1139.46	**0.000**	14.46	4.42	**0.000**
	Female	4272.48	898.51	​	16.87	5.70	​
Sartorius	Male	259.17	51.67	**0.000**	11.30	3.01	**0.001**
	Female	184.02	41.81	​	12.45	3.16	​
ILPS	Male	753.62	180.88	**0.000**	6.80	2.90	0.088
	Female	512.41	106.67	​	6.33	2.19	​
RF	Male	507.46	129.69	**0.000**	8.77	3.16	0.618
	Female	329.59	73.00	​	8.95	2.78	​
PEC	Male	560.96	133.59	**0.000**	7.33	2.85	**0.000**
	Female	365.14	79.70	​	9.22	2.85	​
OR	Male	1240.09	294.49	**0.000**	6.23	2.21	**0.000**
	Female	947.75	280.79	​	7.20	2.22	​
VL	Male	1476.35	457.24	**0.000**	11.59	3.80	0.764
	Female	960.55	314.06	​	11.82	3.94	​

CSA, cross sectional area; IMFF, intramuscular fat fraction; TFL, tensor fasciae latae; GMin, gluteus minimus; GMed, gluteus medius; GMax, gluteus maximus; ILPS, iliopsoas; RF, rectus femoris; PEC, pectineus; OR, internal obturator; VL, vastus lateralis. Bold font indicates P < 0.05.

### 3.2 Age-related differences in CSA and fat infiltration of hip muscles

**FIGURE 3:**
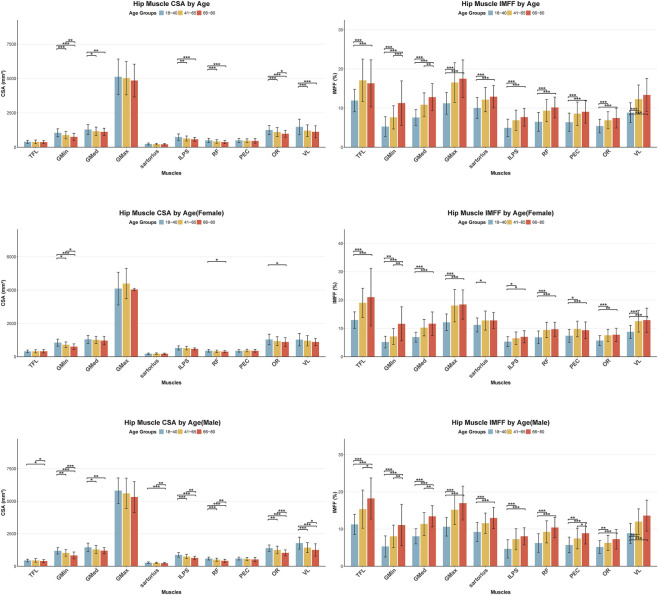
Analysis of age-related effects on CSA and IMFF of hip muscle (*p < 0.05, **p < 0.01, ***p < 0.001). CSA, cross sectional area; IMFF, intramuscular fat fraction; TFL, tensor fasciae latae; GMin, gluteus minimus; GMed, gluteus medius; GMax, gluteus maximus; ILPS, iliopsoas; RF, rectus femoris; PEC, pectineus; OR, internal obturator; VL, vastus lateralis.

Participants were stratified into three age groups (18–40, 41–65, and 66–80 years). Results demonstrated a progressive decline in the CSA of the ten hip muscles with advancing age. Significant differences were observed in GMin, GMed, ILPS, RF, OR, and VL. Although the CSA of the TFL, GMax, sartorius, and PEC also exhibited gradual reductions across age groups, these trends did not reach significance, even in pairwise comparison between the 18–40 and 66–80 years groups. Conversely, the IMFF increased significantly with age in all ten muscles. Pairwise comparisons between age groups (18–40 vs. 41–65 vs. 66–80) revealed smaller differences between the 41–65 and 66–80 cohorts. Sex-stratified analyses indicated that age exerted a more pronounced effect on CSA and IMFF of musles in males than in females (Figure 3).

### 3.3 Laterality effects on CSA and fat infiltration of hip muscles

**FIGURE 4:**
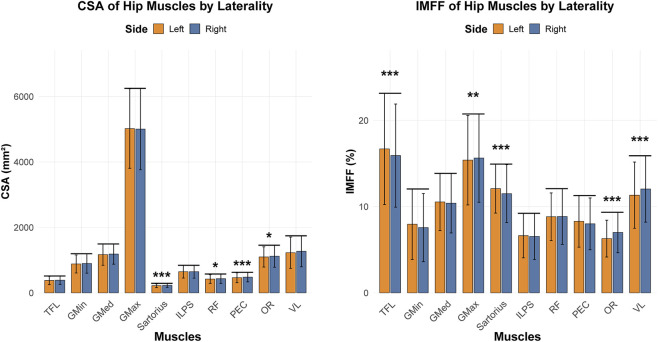
Comparison of CSA and IMFF of hip between the dominant and non-dominant limbs (*p < 0.05, **p < 0.01, ***p < 0.001). CSA, cross sectional area; IMFF, intramuscular fat fraction; TFL, tensor fasciae latae; GMin, gluteus minimus; GMed, gluteus medius; GMax, gluteus maximus; ILPS, iliopsoas; RF, rectus femoris; PEC, pectineus; OR, internal obturator; VL, vastus lateralis.

Laterality had minimal influence on CSA of hip musle, with significant differences observed only in the sartorius, PEC, and OR—all exhibiting larger CSA on the right side. For IMFF, significant laterality differences were detected in the TFL, GMax, sartorius, OR, and VL. Higher IMFF were noted in the left TFL and sartorius, whereas the right GMax, OR, and VL demonstrated greater IMFF (Figure 4).

### 3.4 Correlation analysis

**FIGURE 5:**
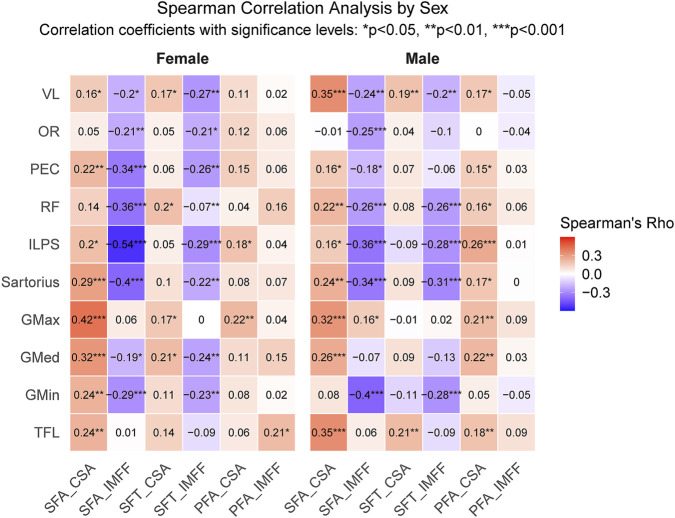
Spearman correlation analysis of hip composition parameters. Heatmap displays Spearman’s correlation coefficients (ρ) between factors (*p < 0.05, **p < 0.01, ***p < 0.001). SFA, subcutaneous fat area; PFA, proximal femoral area; CSA, cross sectional area; IMFF, intramuscular fat fraction; TFL, tensor fasciae latae; GMin, gluteus minimus; GMed, gluteus medius; GMax, gluteus maximus; ILPS, iliopsoas; RF, rectus femoris; PEC, pectineus; OR, internal obturator; VL, vastus lateralis.

After adjusting for sex, most muscles exhibited positive correlations between CSA and SFA (males: TFL, GMed, GMax, sartorius, ILPS, RF, PEC, VL; females: TFL, GMin, GMed, GMax, sartorius, ILPS, PEC, VL). In contrast, IMFF was negatively correlated with SFA (males: GMin, GMax, sartorius, ILPS, RF, PEC, OR, VL; females: GMin, GMed, sartorius, ILPS, RF, PEC, OR, VL). SFT showed weaker correlations with the CSA and IMFF of hip musle than SFA. PFA correlated positively with CSA in most male muscles (except GMin and OR) and selectively in female muscles (GMax, ILPS), but demonstrated negligible associations with IMFF (Figure 5).

### 3.5 Multivariate analysis for IMFF of hip muscles

**FIGURE 6:**
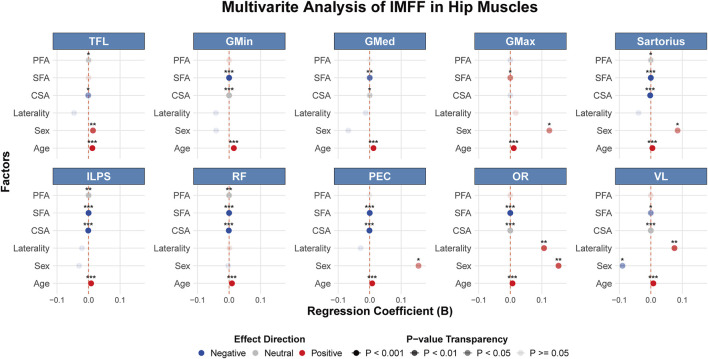
Forest plot of multivariate analysis of IMFF in hip muscles using GEE (*p < 0.05, **p < 0.01, ***p < 0.001). SFA, subcutaneous fat area; PFA, proximal femoral area; IMFF, intramuscular fat fraction; TFL, tensor fasciae latae; GMin, gluteus minimus; GMed, gluteus medius; GMax, gluteus maximus; ILPS, iliopsoas; RF, rectus femoris; PEC, pectineus; OR, internal obturator; VL, vastus lateralis; GEE, generalized estimating equations.

Multivariate analyses were performed to assess determinants of IMFF for each muscle, incorporating age, sex, laterality, corresponding muscle CSA, SFA, and PFA as independent variables (Figure 6). Age showed significant positive associations with IMFF in all ten muscles. The effects of sex and laterality varied by specific muscle. While corresponding muscle CSA and SFA reached statistical significance for most muscles, their effect sizes were clinically negligible. PFA was significant only for a minority of muscles, with similarly trivial effect sizes. These findings underscore age as the predominant factor influencing musculoskeletal metrics.

## 4 Discussion

This study employed the mDIXON QUANT sequence to quantitatively assess the CSA and IMFF of ten hip muscles, Achieving its primary objectives, it established a robust imaging protocol and generated stratified normative reference data for a healthy Asian population. Our work advances the field by providing a more comprehensive anatomical assessment, analyzing a larger cohort, and utilizing a multi-level averaging protocol to reduce measurement bias. The findings elucidate key factors influencing the CSA and IMFF of hip muscles, highlight age as the predominant driver of degeneration, and offer a data foundation with direct implications for clinical practice.

The observed gender differences in CSA and IMFF of hip muscles align with endocrine physiology. Estrogen plays a pivotal role in subcutaneous adipose tissue formation, with studies demonstrating that it enhances the expandability of subcutaneous adipose tissue, thereby promoting preferential fat deposition in females (Mair et al., 2020; Katzer et al., 2021; Johnson et al., 2024). The study demonstrated that males exhibit larger CSA of muscles and less fat deposition compared to females, which aligns with existing research findings (Li J. et al., 2022). Androgens (e.g., testosterone) serve as critical regulators of muscle growth in males. By binding to androgen receptors in muscle tissue, androgens significantly enhance protein synthesis, thereby promoting muscle fiber growth and hypertrophy (Schiaffino et al., 2021; Guilherme et al., 2022).

Sex-based differences in muscle fiber composition may contribute to variations in Intramuscular fat Infiltration. Males typically exhibit a higher proportion of type II muscle fibers (Maher et al., 2009; Horwath et al., 2021; Owoeye et al., 2024; Schlegel and Křehký, 2022; Yang et al., 2022). Due to their lower mitochondrial content and higher oxidative stress, type II fibers are more susceptible to fatty infiltration (Zhu et al., 2024). However, males also possess a greater abundance of satellite cells within type II fibers, which play a key role in muscle regeneration (Yang et al., 2022). This may mitigate fat deposition by enhancing regenerative capacity (Sheptulina et al., 2023). These findings underscore the necessity of sex-specific reference ranges in clinical imaging. Females exhibit lower hip muscle mass and greater fat infiltration, which may elevate the risk of osteoporotic fractures due to reduced mechanical loading on bone. Additionally, fat infiltration may accelerate osteoarthritis progression via pro-inflammatory cytokine secretion. Consequently, rehabilitation strategies for female patients should emphasize resistance training to augment muscle mass, whereas males may benefit more from interventions focused on maintaining muscle strength (Rostron et al., 2022).

Our study demonstrated that age significantly influenced Intramuscular fat infiltration in all ten observed hip muscles, with significant effects on IMFF values. Regarding muscle CSA, no significant differences were observed in the TFL, GMin, sartorius, or PEC in response to age. This suggests that fat infiltration may precede muscle atrophy—a key finding for implementing early interventions. Early targeted exercises to reduce muscle fat content and increase muscle mass are recommended.

The age-related decline in muscle protein synthesis, preferential denervation of type II fast-twitch fibers, and disuse atrophy may contribute to the reduction in hip muscle CSA. Furthermore, increased fat infiltration in muscles with aging may be attributed to pro-inflammatory cytokines (such as IL-6 and TNF-α) promoting adipogenic differentiation of mesenchymal stem cells, insulin resistance, decreased lipid oxidation capacity leading to ectopic fat deposition, and loss of mechanical stress that normally inhibits adipogenesis (Delmonico et al., 2009; Dondero et al., 2024).

Notably, our finding of a significant age-related increase in IMFF contrasts with some prior studies that reported no such change in specific hip muscles (Marcon et al., 2016; Belzunce et al., 2022). This discrepancy may be attributed to several factors. First, population differences may exist; our cohort comprised an Asian population, whereas referenced studies were based on Western cohorts, and genetic, lifestyle, or dietary factors could influence fat infiltration patterns. Second, our measurement protocol used a multi-level average of IMFF, which might capture diffuse, age-related fat accumulation more sensitively than single-slice measurements. Third, our finer age stratification (18–40, 41–65, 66–80 years) could reveal progressive changes that are masked when comparing broader age categories. Segmented analysis showed relatively small differences between the 41–65 and 66–80 years groups, suggesting that intramuscular fat infiltration enters a rapid accumulation phase during middle age, with the rate of increase slowing later, though cumulative effects remain significant. This indicates middle age as a critical window for intervention, emphasizing the need for early management of metabolic risk factors such as obesity and diabetes (Li C. W. et al., 2022).

Analysis stratified by sex and age group revealed that muscle CSA and IMFF were more strongly influenced by age in males than in females. This finding is inconsistent with a previous study (Johnson et al., 2024), which reported a positive correlation between muscle fat infiltration in the lower leg and age that was statistically significant only in females. However, a meta-analysis on lumbar paraspinal degeneration (Dallaway et al., 2020) and a study on thigh muscles (Engelke et al., 2022) highlighted heightened age-related susceptibility to fat infiltration in males. These discrepancies suggest regional and sex-specific variations in muscular degenerative sensitivity.

The CSA of most muscles showed a positive correlation with SFA, while IMFF demonstrated a negative correlation with SFA. In non-obese individuals, moderate energy reserves (subcutaneous fat) may support muscle synthesis (Longo et al., 2021; Helms et al., 2023). Studies on the relationship between thigh muscle CSA and BMI revealed that BMI was positively correlated with thigh muscle CSA, whereas BMI typically correlates with SFA. The positive association between muscle CSA and BMI may reflect overall body size influence, suggesting that individuals with higher BMI tend to possess both greater subcutaneous fat and larger muscle volume (Lang et al., 2010). This observation may explain the positive correlation between muscle CSA and SFA. The positive association between hip SFA and muscle CSA might also be attributed to leptin and adiponectin secreted by subcutaneous fat. Adipocytes secrete leptin, which stimulates muscle growth under normal metabolic conditions (Lu et al., 2023). Another crucial hormone produced by adipose tissue is adiponectin, which signals through adiponectin receptors 1 and 2 (AdipoR1 and AdipoR2). AdipoR1 is predominantly expressed in skeletal muscle and activates the AMPK-SIRT1-PGC-1α axis, thereby inhibiting apoptosis, regulating glucose and lipid metabolism, promoting fatty acid oxidation and glucose uptake in muscle, and maintaining energy homeostasis (Alizadeh Pahlavani, 2022). Additionally, adiponectin enhances myoblast differentiation and fusion, supporting muscle repair (Chen et al., 2023).

The influence of laterality on the CSA of hip muscle was minimal, with significant differences observed only in the sartorius, PEC, and OR, all of which exhibited larger CSAs on the right side compared to the left. Regarding fat infiltration in the hip muscles, differences were noted in the TFL, GMax, sartorius, OR, and VL. Specifically, the left side demonstrated higher IMFF in the TFL and sartorius, whereas the right side exhibited higher IMFF in the GMax, OR, and VL. A previously published study (Belzunce et al., 2021) reported that healthy subjects exhibited higher IMFF in GMAX on the left side compared to the right, which the authors attributed to limb dominance. This finding contrasts with the results of the present study, in which all participants were right-handed. The discrepancy may stem from habitual force exertion patterns, suggesting that laterality effects on hip muscles could be influenced by handedness. Further investigation through targeted movement training is warranted to validate this hypothesis. It should be noted that limb dominance in this study was inferred from self-reported handedness, a pragmatic surrogate in cross-sectional design. We acknowledge that upper limb dominance may not fully reflect lower limb motor patterns, which could influence the interpretation of laterality effects.

Based on our systematic analysis, we recommend using techniques such as mDIXON Quant for quantitative assessment of age-related degeneration of the hip muscle groups before the surgery. The primary variable of interest should be the IMFF, as it directly reflects muscle quality. Rather than applying a universal IMFF threshold, we advocate for age-stratified interpretation: comparing a patient’s preoperative IMFF with the reference ranges established in this study (e.g., means and distributions for 18–40, 41–65, and 66–80 years groups). A markedly elevated IMFF for a given age may indicate accelerated muscle aging, potentially signaling diminished strength, contractile efficiency, and regenerative capacity. Identifying such “accelerated” degeneration preoperatively allows clinicians to anticipate a more challenging recovery and to design targeted prehabilitation strategies (e.g., resistance training, nutritional optimization) aimed at mitigating muscle quality deficits before surgery. Furthermore, postoperative rehabilitation can be personalized based on baseline muscle quality metrics.

## 5 Limitations

This study has several limitations that should be considered. First, due to its cross-sectional design, the observed associations between factors (such as age, sex, and anatomical measurements) and IMFF cannot establish causality. The analysis reveals correlations but does not permit conclusions regarding cause-and-effect relationships. Second, although multivariate analysis was employed to adjust for potential confounders, residual confounding may still be present. Unmeasured variables, such as participants’ physical activity levels, nutritional status, genetic predispositions, could influence muscle composition and were not accounted for in the model. Third, the study population was recruited from a single institution, which may limit the generalizability of our findings. Despite these limitations, this study provides valuable insights into the multifactorial determinants of IMFF in hip muscles and establishes a foundation for future longitudinal or interventional research.

## Data Availability

The original contributions presented in the study are included in the article/supplementary material, further inquiries can be directed to the corresponding author.
